# pH-responsive hyaluronic acid-*co*-poly(acrylic acid) hydrogel scaffolds for modified release of mesalamine; characterization and biosafety evaluation

**DOI:** 10.1039/d5ra08622a

**Published:** 2026-01-26

**Authors:** Huma Liaqat, Muhammad Usman Minhas, Syed Faisal Badshah, Kashif Barkat, Samiullah Khan, Shivani Chopra, Hitesh Chopra, Esmael M. Alyami, Amira Metouekel, Atrsaw Asrat Mengistie, Gehan M. Elossaily, Mudassar Shahid

**Affiliations:** a Faculty of Pharmacy, The University of Lahore Lahore Pakistan pharmacisthuma123@gmail.com kashif.barkat@pharm.uol.edu.pk; b College of Pharmacy, University of Sargodha Sargodha Pakistan us.minhas@hotmail.com; c Faculty of Medical and Health Sciences, Department of Pharmacy, University of Poonch Rawalakot Azad Jammu and Kashmir Pakistan faisal.badshah@upr.edu.pk; d Faculty of Health Sciences, Equator University of Science and Technology Masaka City Uganda; e College of Pharmacy, Margalla Institute of Health Sciences Islamabad Pakistan sami_pharmacist99@hotmail.com; f Department of Biosciences, Saveetha School of Engineering, Saveetha Institute of Medical and Technical Sciences Chennai - 602105 Tamil Nadu India shanuvashisht2730@gmail.com; g Centre for Research Impact & Outcome, Chitkara College of Pharmacy, Chitkara University Rajpura 140401 Punjab India chopraontheride@gmail.com; h Department of Biology, College of Science, King Khalid University PO Box 960 Abha 61421 Asir, Saudi Arabia ealshahi@kku.edu.sa; i University of Technology of Compiègne EA 4297 TIMR 60205 Compiègne Cedex France amira.m@bioval-oi.com; j Department of Biology, Bahir Dar University P.O.Box 79 Bahir Dar Ethiopia Atrsawresearcher@gmail.com; k Department of Pharmaceutics, College of Pharmacy, King Saud University Saudi Arabia mahmad1@ksu.edu.sa; l Department of Basic Medical Sciences, College of Medicine, AlMaarefa University Diriyah 13713 Riyadh Saudi Arabia jabdelmenam@um.edu.sa; m Research Center, Deanship of Scientific Research and Post-Graduate Studies, AlMaarefa University Diriyah 13713 Riyadh Saudi Arabia; n Health And Medical Research Centre (HMRC), King Khalid University Abha 61413 Saudi Arabia

## Abstract

The current study focuses on the development of pH-sensitive hydrogels for the controlled release of mesalamine at a specific pH. A free radical polymerization technique was used, and the formulated hydrogels were characterized by Fourier transform infrared spectroscopy (FTIR), thermogravimetric analysis, scanning electron microscopy, swelling and drug release studies. FTIR confirmed the successful formation of a polymeric network with efficient incorporation of the drug. TGA revealed that the hydrogel demonstrated significantly improved thermal stability compared to the unmodified polymer, with SEM images showing a rough surface with a notable porous structure. Swelling and drug release behaviors were assessed at 37 °C in buffer solutions simulating gastric (pH 1.2) and intestinal (pH 7.4) conditions. The results demonstrated significantly higher swelling ratios and drug release rates at pH 7.4, indicating pH-responsive characteristics of the formulation, favoring enhanced drug release in the intestinal conditions. Increasing the concentrations of hyaluronic acid and acrylic acid led to enhanced porosity, improved swelling behavior, increased drug loading capacity, faster release rates, and a higher gel fraction, while simultaneously reducing the sol fraction. These observations suggested that the developed pH-sensitive hydrogel holds considerable potential as an effective carrier for pH-responsive, site-specific drug delivery systems. This study highlights the significant advancements in the design of hydrogels with tailored properties for controlled drug release, offering new opportunities for precision medicine.

## Abstract 2

The current study focuses on the development of pH-sensitive hydrogels for the controlled release of mesalamine at a specific pH.

## Graphical abstract

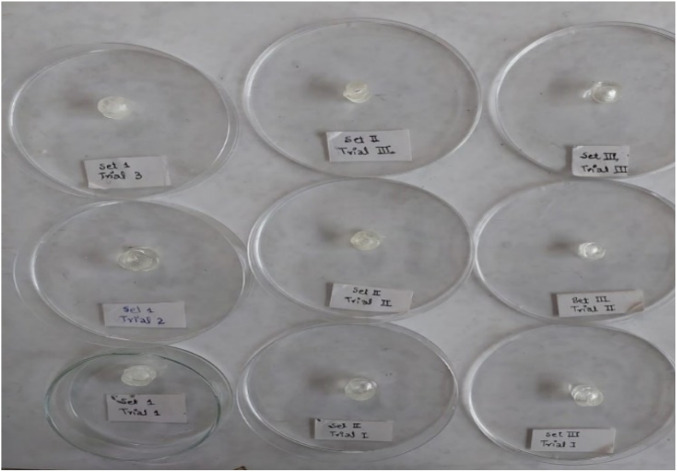

## 1. Introduction

Controlled-release drug delivery systems are distinguished from conventional formulations by their ability to modulate the release rate of the drug over time, rather than rapid and uncontrolled release. These systems integrate advanced designs to regulate the drug release, thereby providing a more sustained and predictable therapeutic effect.^1^ In recent decades, controlled drug release systems (CDRSs) have garnered significant attention due to their capacity to enhance the efficacy of existing medications. These systems are engineered to regulate the kinetics of drug release, thus enhancing therapeutic efficacy while concurrently minimizing undesirable side effects.^1,2^ Conventional drug delivery systems often result in high initial plasma concentrations, which are followed by a rapid decline due to drug elimination, leading to fluctuating drug levels, frequent dosing, and an increased risk of toxicity. In contrast, CDRSs are designed to regulate the release of the drug, addressing these challenges by modulating release kinetics and minimizing issues such as short half-lives, nonspecific distribution, and off-target toxicity. CDRSs are particularly useful for treating chronic conditions that require extended therapeutic treatment. Advances in understanding disease mechanisms and the development of targeted drug delivery systems have led to the creation of new dosage forms and carrier systems with improved controlled release properties. These developments allow for precise drug delivery to targeted sites by utilizing the specific characteristics of the disease and the system itself.^3^

Among these advancements, hydrogel-based systems stand out due to their remarkable effectiveness. These systems enhance therapeutic outcomes by prolonging the duration of action, reducing the frequency of administration, stabilizing plasma drug concentrations, and minimizing side effects. Moreover, site-specific drug delivery systems enhance the accumulation of the therapeutic agent at the intended pathological site, thereby minimizing systemic exposure, reducing off-target cytotoxicity, and improving the overall therapeutic index, which is particularly beneficial in the long-term pharmacological management of chronic conditions.^4^ Hydrogels are distinguished by a three-dimensional polymeric framework that has the ability to retain substantial amounts of water. The stability of these networks is maintained through crosslinking, which strengthens the system by inhibiting dissolution and ensuring structural integrity through both physical and chemical interactions.^5^ The growing focus on personalized medicine and precision therapies has driven the advancement of smart biomaterials, such as innovative hydrogels. These hydrogels are designed to respond to specific physiological stimuli, enabling customized therapeutic approaches and improving the effectiveness of precision treatments.^6^ Stimulus-responsive hydrogels are advanced biomaterials designed to release drugs in response to various external stimuli, including pH, temperature, electrical and magnetic fields, and light. These hydrogels exhibit dynamic behavior, enabling controlled drug delivery based on specific environmental changes.^7^ Smart hydrogels exhibit significant alterations in their macroscopic and physical properties, including swelling behavior and mechanical strength. These changes allow for the precise regulation of drug release and enhance the therapeutic efficacy of the system.^8^ These advanced drug delivery platforms significantly reduce dosing frequency by sustaining therapeutic plasma concentrations from a single administration, while concurrently attenuating systemic adverse effects through spatially controlled and site-specific drug release mechanisms.^9^

Crohn's disease can affect any part of the gastrointestinal tract with transmural involvement, while ulcerative colitis is confined to the mucosal layer of the colon and rectum. Genetic predisposition, including variants in genes related to immune function and intestinal barrier integrity, increases susceptibility to these conditions. Environmental factors such as diet, microbiome alterations, and smoking can exacerbate IBD, while dysfunction of the intestinal barrier allows microbial antigens to penetrate the mucosa, further aggravating the inflammatory response. Patients with these conditions typically require extended pharmacological treatment to initiate and maintain remission. These therapeutic approaches contribute to reducing the risk of disease recurrence and minimize the dependence on corticosteroids, prolonged hospitalization, and surgical procedures.^10^

Over the past thirty years, mesalamine (5-aminosalicylic acid, 5-ASA) has remained the cornerstone pharmacological agent in the frontline management of ulcerative colitis, owing to its established efficacy in inducing and maintaining mucosal remission. Mesalamine exerts its therapeutic effects in inflammatory bowel disease (IBD) through its local anti-inflammatory action, targeting the inflamed mucosal tissue of the gastrointestinal tract. It inhibits the production of pro-inflammatory cytokines and leukotrienes, thereby reducing inflammation and promoting mucosal healing. Additionally, mesalamine may modulate the immune response by influencing immune cell activity and inflammatory mediators, contributing to a more balanced immune environment within the gut. It also helps preserve intestinal barrier function by mitigating inflammation and supporting epithelial repair. Overall, mesalamine is crucial in managing IBD, facilitating remission, and enhancing patient outcomes and quality of life.^11^ Standard treatments for Crohn's disease and ulcerative colitis include 5-aminosalicylic acid, corticosteroids, sulfasalazine, thiopurines, and methotrexate. Among the available therapeutic modalities, mesalamine is extensively regarded as a well-tolerated and efficacious first-line agent for the treatment of ulcerative colitis, demonstrating favorable safety profiles across numerous clinical studies. Research has shown that high doses of mesalamine, whether administered orally, rectally, or in combination, can effectively manage active ulcerative colitis. Furthermore, mesalamine has been shown to offer protective effects against the development of colorectal cancer.^12^ Oral mesalamine is quickly absorbed in the upper intestine, leading to reduced effectiveness for IBD due to low colon concentration. Targeted release strategies aim to improve its delivery and efficacy in the colon.^13^

Biopolymers are favored over synthetic polymers for the development of self-healing poly-complexes due to their superior biocompatibility and non-toxic properties. Hyaluronic acid (HA) is a naturally occurring, high-molecular-weight, linear polysaccharide classified as a glycosaminoglycan, composed of repeating disaccharide units comprising β-(1,4)-linked d-glucuronic acid and β-(1,3)-linked *N*-acetyl-d-glucosamine. As a weak polyanion with a p*K*_a_ of 2.9, HA demonstrates remarkable biocompatibility, viscoelasticity, and non-toxicity. Additionally, its strong hydrophilic nature, along with its lubricating and moisturizing properties, further enhances its biocompatibility and plays a role in preventing biofilm formation.^14^ Hyaluronic acid is quite a recent addition to the category of polysaccharides under consideration for employment in the development of self-healing materials. The molecular weight of HA exhibits considerable variability, ranging from approximately 5000 to 2 000 000 Da. To enhance its mechanical stability, HA is frequently chemically modified through cross-linking with various agents, resulting in the formation of robust hydrogels. Owing to its inherent biocompatibility, pronounced hydrophilicity, and capacity to form self-healing, injectable hydrogel matrices, hyaluronic acid has emerged as a highly promising candidate for drug delivery applications, particularly in the treatment of inflammatory bowel disease. As a natural component of the extracellular matrix, HA is well-tolerated and supports enhanced biocompatibility in biomedical applications. Its high molecular weight and dynamic properties, such as those facilitated by reversible cross-linking, allow for the development of hydrogels that can encapsulate and release therapeutic agents in a controlled manner.^15^ The advancement of hyaluronic acid (HA)-based hydrogels presents a significant opportunity for the management of inflammatory bowel disease (IBD). HA is well-regarded for its anti-inflammatory properties, which are advantageous in addressing IBD, a disorder marked by persistent inflammation of the gastrointestinal tract. HA's capacity to modulate inflammatory responses and facilitate tissue repair aligns with the therapeutic objectives for IBD treatment.

Recent developments in controlled-release systems aim to improve targeted drug delivery to the colon for inflammatory bowel disease. The integration of mesalamine, a well-established anti-inflammatory agent for IBD, into HA-based hydrogels offers the potential for a synergistic therapeutic effect. While mesalamine directly alleviates inflammation within the colon, HA's anti-inflammatory and regenerative properties can enhance this effect. By embedding mesalamine within HA-based hydrogels, it is possible to achieve targeted and sustained drug delivery to inflamed intestinal regions, thereby potentially increasing therapeutic efficacy and reducing systemic side effects. This combined approach promises a more comprehensive treatment strategy for IBD, effectively addressing both the inflammatory symptoms and the underlying pathophysiology of the disease. The study aims to design a novel polymeric hydrogel scaffold for the controlled release of mesalamine, enhancing its effectiveness. This new system aims to optimize drug delivery, reduce dosage frequency, and enhance patient compliance and safety. In our previous research, similar study was conducted with the utilization of two monomers and the effect of combination of the two monomers was evaluated. In current study, a single monomer has been used and its effect on the different parameters has been examined.

## 2. Materials and methods

### 2.1. Materials

The chemicals used in this study, including sodium hydroxide, potassium chloride, ammonium persulfate, potassium dihydrogen phosphate, 5-aminosalicylic acid, hyaluronic acid, acrylic acid, *N*,*N*-methylenebisacrylamide, ethanol, hydrochloric acid, and sodium hydroxide, were procured from Sigma-Aldrich Chemie GmbH, Steinheim, Germany. All chemicals were of analytical grade. Freshly distilled water, supplied by the research laboratory at the University of Lahore, was used for the experiments.

### 2.2 Methods

#### 2.2.1. Fabrication of hydrogels

Hydrogels were synthesized using a free radical polymerization method, following established protocols from previous research.^16^ A predetermined quantity of hyaluronic acid polymer was accurately weighed and introduced into a beaker containing a specified volume of distilled water as the solvent. The mixture was subjected to continuous agitation at 80 rpm (rpm) for a duration of 30 minutes using a magnetic stirrer to ensure complete homogeneity. Acrylic acid (monomer) was introduced dropwise into the polymer solution, with stirring maintained for 10 minutes. Concurrently, a reaction initiator solution was prepared by dissolving ammonium persulfate (APS) in water. The concentrations of the reactants used in the hyaluronic acid-based hydrogels are detailed in Table 1. The ammonium persulfate (APS) solution was subsequently introduced into the polymer-monomer mixture and stirred continuously for 10 minutes to achieve a uniform and homogenous dispersion. A specified amount of methylene bisacrylamide (MBA) was dissolved in water under continuous stirring until the solution became clear and homogenous. The polymer, monomer, and initiator mixture were then combined with the MBA solution and stirred for an additional five minutes. The resulting reaction mixture was transferred to pre-labeled test tubes and placed in a preheated water bath, initially set at 55 °C for one hour, followed by a temperature increase to 60 °C for an additional two hours.

**Table 1:** Concentration of ingredients in HA-based hydrogel formulations

Formulation code	The concentration of polymer, monomer, crosslinker, and initiator			
	Hyaluronic acid (g)	Acrylic acid (g)	MBA (g)	APS (g)
HA-1	0.05	3	0.04	0.2
HA-2	0.1	3	0.04	0.2
HA-3	0.15	3	0.04	0.2
HA-4	0.1	3	0.04	0.2
HA-5	0.1	4	0.04	0.2
HA-6	0.1	5	0.04	0.2
HA-7	0.1	3	0.04	0.2
HA-8	0.1	3	0.05	0.2
HA-9	0.1	3	0.06	0.2

#### 2.2.2. Washing and cutting

**Fig. 1:**
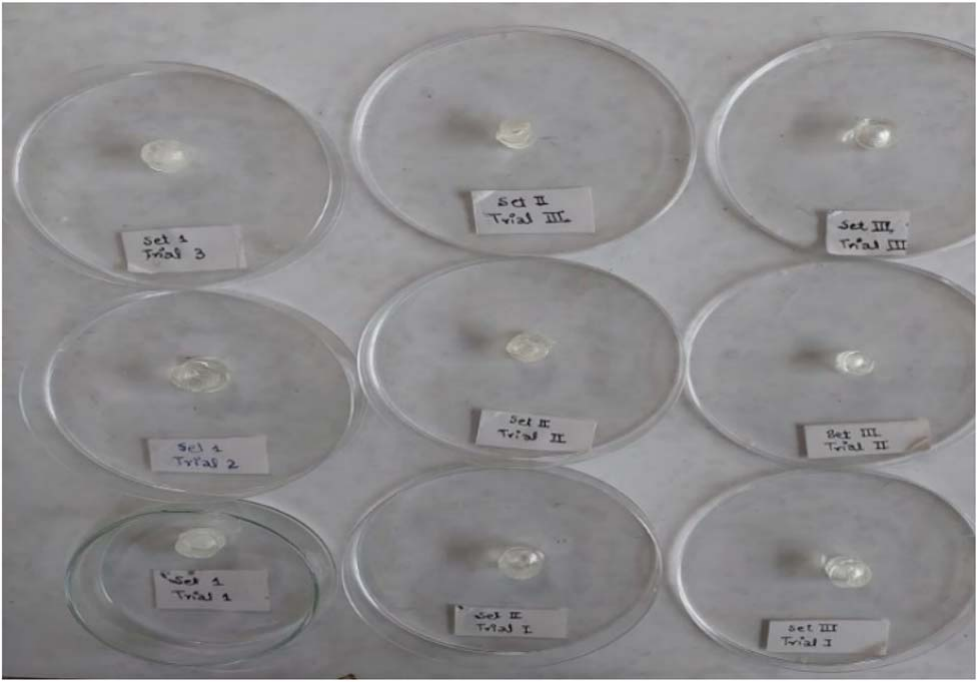
Physical appearance of dried hydrogel discs.

The test tubes containing the hydrogel were removed from the water bath and allowed to cool to room temperature. The formed solid cylindrical hydrogels were carefully removed from the test tubes and accurately sectioned into discs, each measuring 9 mm in thickness. These discs were thoroughly rinsed with a 50 : 50 (v/v) mixture of water and ethanol to eliminate any residual impurities. Finally, these discs were placed in Petri dishes and dried for one week in a vacuum oven at 40 °C to obtain dried hydrogels. The dried hydrogel discs can be observed in Fig. 1.

## 3. Characterization

### 3.1. Fourier transform infrared spectroscopy (FTIR)

FTIR analysis was employed to investigate the diverse interactions between the drug and polymer, evaluate their compatibility, and confirm the establishment of polymeric networks. Both the initial raw materials and the synthesized hydrogels were independently examined using FTIR spectroscopy to identify distinct spectral changes indicative of cross-linking processes essential for successful hydrogel formation.^17^ The hydrogel discs were subjected to grinding to achieve a uniform particle size distribution. The resulting powder was carefully sieved using No. 40 mesh sieve to remove any agglomerates or oversized fragments.^18^ The samples were meticulously deposited onto the surface of a zinc selenide (ZnSe) crystal to ensure optimal contact for spectral analysis. The sample–crystal interface was methodically rotated to maximize the interaction between the evanescent infrared beam and the sample, thereby enhancing the sensitivity of the measurement. Subsequent characterization was performed using attenuated total reflectance Fourier transform infrared (ATR-FTIR) spectroscopy, allowing for comprehensive identification of molecular interactions, crosslinking efficiency, and chemical compatibility within the hydrogel matrix. The Fourier transform infrared spectra were collected within the wavenumber range of 4000 to 400 cm^−1^.

### 3.2. Thermal analysis

The thermal stability of hydrogel formulations and pure reactants was assessed using the TGA module of the Q5000 series thermal analysis system. Hydrogel samples were crushed, sieved, and analyzed (5–10 mg) in a nitrogen environment, with a heating rate of 20°C min^−1^ up to 500 °C. Weight changes were monitored as the temperature increased.^19^

### 3.3. Scanning electron microscopy

The surface morphology of the mesalamine-loaded hydrogels was investigated using an SEM (JEOL Japan Mode: J8M 6480LV). Dried hydrogel discs were cleaved, and they were transferred to a double-adhesive tape affixed to an aluminum stub. Before imaging, the stubs were coated with a layer of gold in an argon atmosphere through a gold sputter module in a high-vacuum evaporator.^20^ The analysis of surface configurations was best accomplished using SEM. The samples were scanned at various resolutions, and micrographs were recorded to visualize the surface morphology.^19^

### 3.4. Powder X-ray diffraction analysis

The amorphous structure of the delivery system primarily favors enhanced aqueous solubility and profound bioavailability.^21^ The crystalline and amorphous characteristics of the pure drug, polymer, and drug-loaded hydrogel formulations were systematically evaluated through powder X-ray diffraction (PXRD) analysis. Powdered samples were carefully loaded into a plastic sample holder, and the surface was meticulously flattened using a glass slide to ensure uniform sample distribution and minimize scattering artifacts. Diffraction patterns were recorded employing a copper Kα radiation source (*λ* = 1.542 Å), utilizing 1 mm divergence and receiving slits to optimize resolution. Samples were scanned across a 2*θ* angular range of 5° to 50°, at a controlled scanning speed of 1° 2*θ* per minute, enabling detailed assessment of the degree of crystallinity and phase transitions within the hydrogel matrix.

### 3.5. Sol–gel fraction

A sol–gel study was performed to determine the uncrosslinked fraction of the gel structure. Hydrogel discs were extracted using the Soxhlet method with distilled water at 80 °C for four hours, then dried in a vacuum oven at 40 °C until a consistent weight was achieved.^22^ The percentage sol and gel fractions were calculated using the equations below.
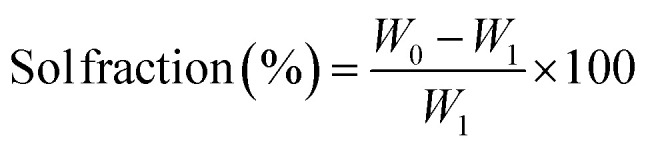

Gel fraction (%) = 100 − Sol fraction

*W*_0_ denotes the initial weight of the hydrogel before Soxhlet extraction, and *W*_1_ symbolizes the weight of the dried hydrogel after Soxhlet extraction.

### 3.6. Swelling studies

Swelling behavior of the hydrogels was systematically evaluated to elucidate their responsiveness to variations in pH. Dried hydrogel discs of known weight were immersed in 50-mL buffers provided in clearly labeled containers.^23^ At predetermined intervals, the hydrogel discs were meticulously retrieved from their respective swelling media to ensure consistency in sampling. Each disc was then gently blotted using absorbent, lint-free material to remove any adhering surface solvent, thereby preventing overestimation of swelling. Subsequently, the discs were promptly weighed using an analytical balance with high precision to accurately quantify the equilibrium swelling capacity. This process was repeated across multiple time points to generate a comprehensive swelling profile, enabling assessment of the hydrogel's responsiveness and stability under varying pH conditions.
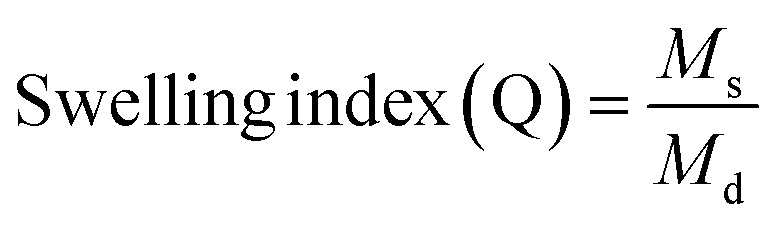
where *M*_d_ is the initial weight of the dried hydrogel, while *M*_s_ is the weight of the swollen hydrogel disc at time *t*.

### 3.7. Polymer volume fraction

The polymer volume fraction, given by *v*_2_, *s*, the amount of polymer that was fully swollen. At pH 1.2 and 7.4, the volume fraction of the polymer in an entirely swollen condition was estimated using equilibrium volume swelling (*V*_eq_). The following equation was used to calculate *V*_2_.
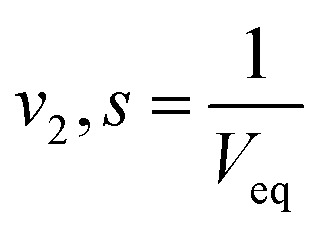


### 3.8. Percent water content

Hydrogels are hydrophilic materials capable of absorbing significant amounts of solvent, which can influence various properties, including the drug concentration within the polymeric network. To determine the actual ratios, the percent water content of the hydrogels was measured. For this, moist hydrogels were placed in pre-weighed Petri dishes and dried in an oven at 110 °C ± 5 °C for 24 hours. After drying, blotting paper was used to confirm the removal of any residual moisture from the hydrogel surfaces. Once fully dried, the Petri dishes were removed from the oven, covered with lids, and placed in a desiccator to cool. The dishes were then reweighed after cooling and removal from the desiccator.^24^ The following equation was used to calculate the water content of hydrogels.

*w* = [(*M*_cms_ − *M*_cds_)/(*M*_cds_ − *M*_c_)] × 100 = (*M*_w_/*M*_s_) × 100

where; *w* = water content, %, *M*_cms_ = mass of container and moist specimen in grams, *M*_cds_ = mass of container and oven dry specimen in grams, *M*_c_ = mass of container in grams, *M*_w_ = mass of water (*M*_w_ = *M*_cms_ − *M*_cds_) grams, *M*_s_ = mass of oven-dry specimen (*M*_s_ = *M*_cds_ − *M*_c_) grams.

### 3.9. Drug loading

**Fig. 2:**
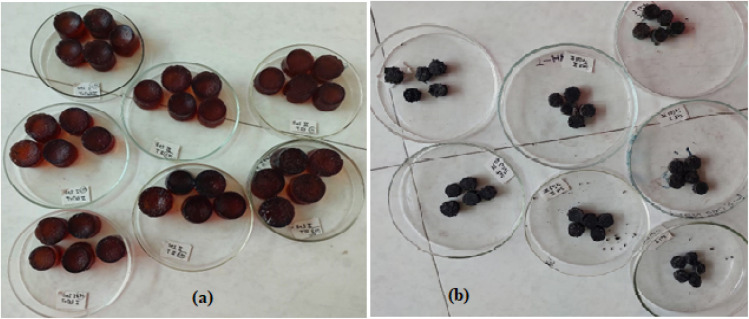
Drug-loaded hydrogel discs (a); dried hydrogel discs (b).

Mesalamine was incorporated into the hydrogel discs employing a combined swelling and diffusion loading technique. The preformed hydrogel discs were submerged in a 1% (w/v) mesalamine solution prepared in phosphate buffer at pH 7.4, maintained under controlled conditions for a duration of five days to allow sufficient drug uptake. Throughout this period, the hydrogels reached equilibrium swelling, facilitating the diffusion of mesalamine molecules into the polymeric network. Upon completion of the loading phase, the discs were carefully withdrawn from the drug solution and gently blotted with absorbent material to eliminate residual surface liquid. To further ensure the removal of any loosely adhered drug molecules on the surface, the discs were rinsed thoroughly with distilled water. This protocol ensured uniform drug entrapment within the hydrogel matrix, optimizing subsequent release characteristics, as depicted in Fig. 2.

The discs were then dried in a vacuum oven at 40 °C until they reached a constant weight.^25^ The given equation was employed to calculate the amount of drug entrapped in the hydrogels.

Drug loaded quantity = *D*_L_ − *D*_UL_

*D*_L_ represents the mass of drug-loaded hydrogel discs, while *D*_UL_ indicates the mass of the unloaded hydrogel discs.

### 3.10. Percent drug-loaded contents

The drug-loaded hydrogel discs were meticulously pulverized using a dry, sterile pestle and mortar to facilitate accurate quantification of the encapsulated mesalamine. The resulting powdered hydrogel samples were accurately weighed and subsequently immersed in 500 mL of phosphate buffer solution (pH 7.4) maintained at 37 °C ± 0.5 °C to simulate physiological conditions. The samples were allowed to incubate for a period of 24 hours to ensure complete drug leaching from the polymeric matrix into the dissolution medium. Following the incubation, the suspension was subjected to centrifugation at 3000 rpm to separate the insoluble hydrogel residues from the supernatant containing the dissolved drug. This procedure enabled precise measurement of drug content by isolating the released mesalamine for further analytical quantification.^19^ The supernatant was carefully collected and subsequently filtered using a 0.45 µm filter paper after centrifugation. Mesalamine content was quantified using a UV spectrophotometer, with measurements taken at a maximum wavelength of 218 nm. The percentage of drug-loaded content was determined using the following formula.
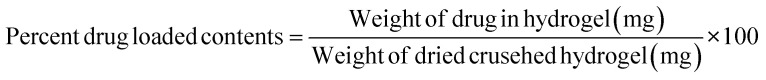


### 3.11. *In vitro* release studies

To determine how the pH influenced drug release, *in vitro* drug release studies were conducted employing USP dissolution apparatus II (DIS-08S).^26^ Prior to immersion in 900 mL of dissolution media at pH 1.2 and pH 7.4, the hydrogel discs were precisely weighed to enable accurate assessment of the pH-dependent mesalamine release profile. The dissolution apparatus was maintained at a physiological temperature of 37 °C, with the paddle rotation speed set at 50 rpm to closely simulate gastrointestinal motility. At predetermined time intervals, 5 mL aliquots were withdrawn from the dissolution medium for analysis. Each collected sample underwent triplicate spectrophotometric evaluation using UV-visible spectroscopy at an absorption maximum (*λ*_max) of 218 nm. To maintain sink conditions and constant volume throughout the experiment, an equal volume of fresh pre-warmed buffer was immediately replenished following each sampling. This methodology ensured reliable and reproducible data reflecting the controlled drug release behavior under varying pH conditions.

### 3.12. Biocompatibility evaluation

Biocompatibility evaluations were conducted using six healthy New Zealand White NZW rabbits, both male and female, aged 08–10 weeks, which were randomly assigned into control and experimental cohorts to assess the safety profile of the synthesized hydrogels. This study was duly authorized by the Institutional Research Ethics Committee (IREC) of the Faculty of Pharmacy, The University of Lahore, Pakistan, under reference number IREC-2023-60, ensuring compliance with all established ethical standards for the care and use of laboratory animals. The experimental group received an oral administration of the developed hydrogels at a dose of 2 g kg^−1^ body weight, while the control group was maintained solely on standard food and water without any treatment. Following a 14-day observation period, blood samples were systematically collected *via* venipuncture for comprehensive hematological analysis to detect any systemic toxicological effects. Subsequently, the animals were humanely euthanized in accordance with ethical guidelines, and major organs—including liver, kidney, heart, and spleen—were harvested and fixed in 10% formalin solution. These tissues were then subjected to detailed histopathological examination to identify any morphological changes or adverse tissue reactions attributable to the hydrogel administration.^27^

### 3.13. Stability studies

Stability studies of the drug-loaded hydrogels were carried out following ICH guidelines and established protocols. Hydrogels were sealed in airtight glass containers and stored in a stability chamber (Memmert Beschickung, Japan) at 40 ± 2 °C and 75 ± 5% relative humidity for 6 months. Samples were evaluated at 0, 3, and 6 months for physical appearance to detect any morphological changes, FTIR spectroscopy to assess potential chemical alterations in the polymer network, and drug release profiles to monitor the sustained release of 5-ASA under the specified storage conditions.

## 4. Results and discussion

### 4.1. Fourier transform infrared (FTIR) spectroscopy

**Fig. 3:**
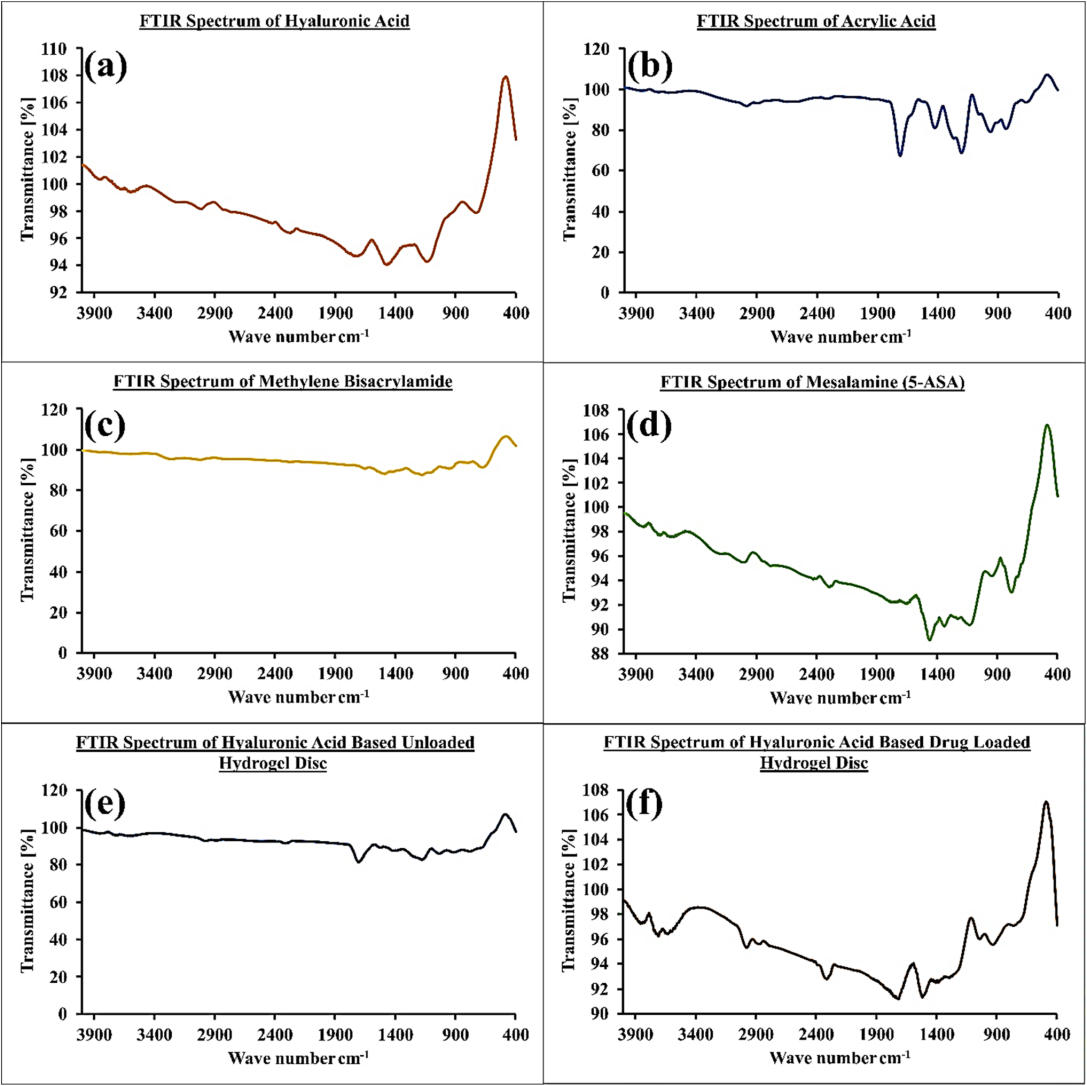
Polymer (a), monomer (b), and crosslinker (c), drug (d), unloaded hydrogel (e), and loaded hydrogel (f).

FTIR spectra of individual ingredients and combinations are exhibited in Fig. 3. Based on hyaluronic acid spectral data from FTIR, the strong band close to 3250 cm^−1^ was related to hydrogen bonds of O–H and N–H and vibrations for stretching of the *N*-acetyl side chain. Due to the C–H stretching vibrations, a collection of overlapping bands of moderate intensity was visible around 2972 cm^−1^. The asymmetric (C=O) and symmetric (C–O) stretching modes of the planar carboxyl groups in hyaluronate are responsible for the bands at 1708 cm^−1^ and 1452 cm^−1^.^28^ The FTIR spectrum of acrylic acid (AAc) exhibits characteristic absorption bands indicative of its molecular structure. Prominent peaks observed at 2981 cm^−1^ correspond to the asymmetric and symmetric stretching vibrations of the methylene (–CH_2_^−^) groups. The presence of a strong peak at 1707 cm^−1^ is attributed to the stretching vibration of the carbonyl (C=O) group within the carboxylic acid moiety. Additionally, bands appearing at 1240 cm^−1^ and 1193 cm^−1^ are assigned to O–H bending and C–H bending vibrations, respectively, further confirming the hydroxyl functionalities and alkyl backbone. A distinct absorption at 655 cm^−1^ corresponds to the out-of-plane bending associated with the carbonyl group. In the case of methylene bisacrylamide (MBA), the FTIR spectrum displays characteristic peaks that confirm the presence of amide functionalities and vinyl groups. The broad band centered around 3250 cm^−1^ is indicative of N–H stretching vibrations from amide groups. The strong absorption at 1641 cm^−1^ corresponds to the C=O stretching vibration characteristic of the amide I band. Furthermore, the peak at 1483 cm^−1^ is attributed to C=C stretching vibrations from the acrylamide vinyl groups. These spectral features collectively validate the molecular components and functional groups present in the crosslinker.^29^ In the FTIR spectrum of methylene bisacrylamide (MBA), a characteristic peak corresponding to the C–N stretching vibration was identified at 1288 cm^−1^, consistent with previously reported data.^30^ This peak further confirms the presence of amide linkages within the crosslinker structure. Regarding mesalamine, its FTIR spectrum revealed distinct absorption bands indicative of key functional groups. Peaks observed at 1647 cm^−1^ and 1571 cm^−1^ were attributed to the stretching vibrations of the carboxylic acid (COOH) and amino (NH_2_) groups, respectively. Additionally, bands at 1338 cm^−1^ and 1242 cm^−1^ corresponded to the C–N stretching and O–H bending vibrations, further validating the molecular structure of mesalamine and its characteristic functional groups.^25^

The chemical interactions among the hydrogel constituents—hyaluronic acid (HA), methylene bisacrylamide (MBA), acrylic acid (AAc), and ammonium persulfate (APS)—resulted in noticeable shifts in their characteristic FTIR absorption peaks, providing evidence for successful cross-linking and polymerization processes. Specifically, alterations in the spectral bands corresponding to HA, AAc, and MBA indicated the establishment of a novel polymeric network structure. The disappearance of the characteristic C=C stretching vibrations further corroborated the completion of graft copolymerization between HA and AAc. Furthermore, the loading of mesalamine into the hydrogel matrix was substantiated by the observed peak shifts in the FTIR spectra, which suggest enhanced hydrogen bonding interactions and confirm the efficient incorporation of the drug within the hydrogel network.^25^ The changes in mesalamine's characteristic peak bands confirm its successful integration into HA-AAc gels, resulting in the formation of the HA-AAc-MSN polymeric system.

### 4.2. Thermogravimetric analysis (TGA)

**Fig. 4:**
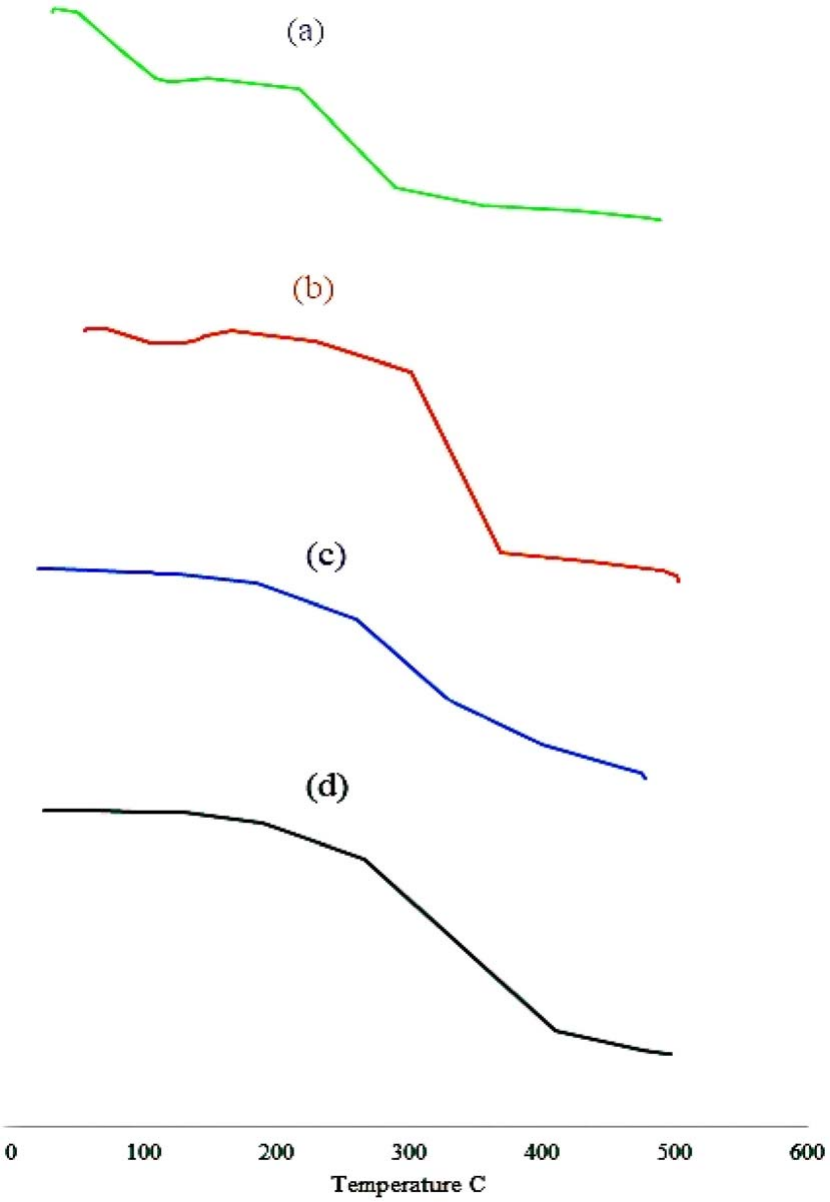
Polymer (a), unloaded hydrogel (b), drug (c), and loaded hydrogel (d).

TGA investigations were performed to assess the thermal stability of hyaluronic acid, mesalamine, unloaded, and loaded hydrogels, determining sample decomposition characteristics after heating. Fig. 4 depicts thermograms of HA, mesalamine, unloaded, and loaded hydrogel discs from 0 to 500 °C. The thermogravimetric analysis (TGA) thermogram of hyaluronic acid (HA) exhibited an initial weight loss in the temperature range of 44 °C to 145 °C, which is attributed to the evaporation of adsorbed and bound water molecules. A subsequent weight reduction observed between 290 °C and 434 °C corresponds to the onset of partial thermal degradation and breakdown of the polymer's molecular structure. The fundamental structural framework of HA disintegrated at 488 °C to 498 °C, and the weight loss was attributable to the dissociation of C–O–C bonds and proceeded until complete degradation.^31^

The thermogram of mesalamine exhibited a distinct endothermic peak between 285 °C and 355 °C, corresponding to its melting point. In the physical mixtures of mesalamine and hyaluronic acid (HA), this peak appeared within a range of 266 °C to 409 °C. Similarly, the TGA thermogram of the hydrogel showed a slight weight loss of 2–3% at 190 °C, attributed to the loss of water from the polymers. A significant weight loss of 75% was observed as the temperature increased to 475 °C, indicating the onset of hydrogel degradation, which continued until complete decomposition. The TGA thermograms of the individual reactants compared to the synthesized hydrogel indicate that the processes of cross-linking and grafting markedly enhanced the thermal stability of hyaluronic acid, as evidenced by a higher degradation onset temperature and reduced weight loss at elevated temperatures. When compared to the unreacted hyaluronic acid, the resulting hydrogel exhibited enhanced thermal stability, which can be attributed to the increased intermolecular interactions induced by cross-linking, grafting, and polymerization.^25^ Bucak and colleagues developed alginate-based hydrogel beads and observed an enhancement in the thermal stability of the hydrogel beads compared to the raw material, which supports our findings.^32^

### 4.3. Scanning electron microscopy (SEM)

**Fig. 5:**
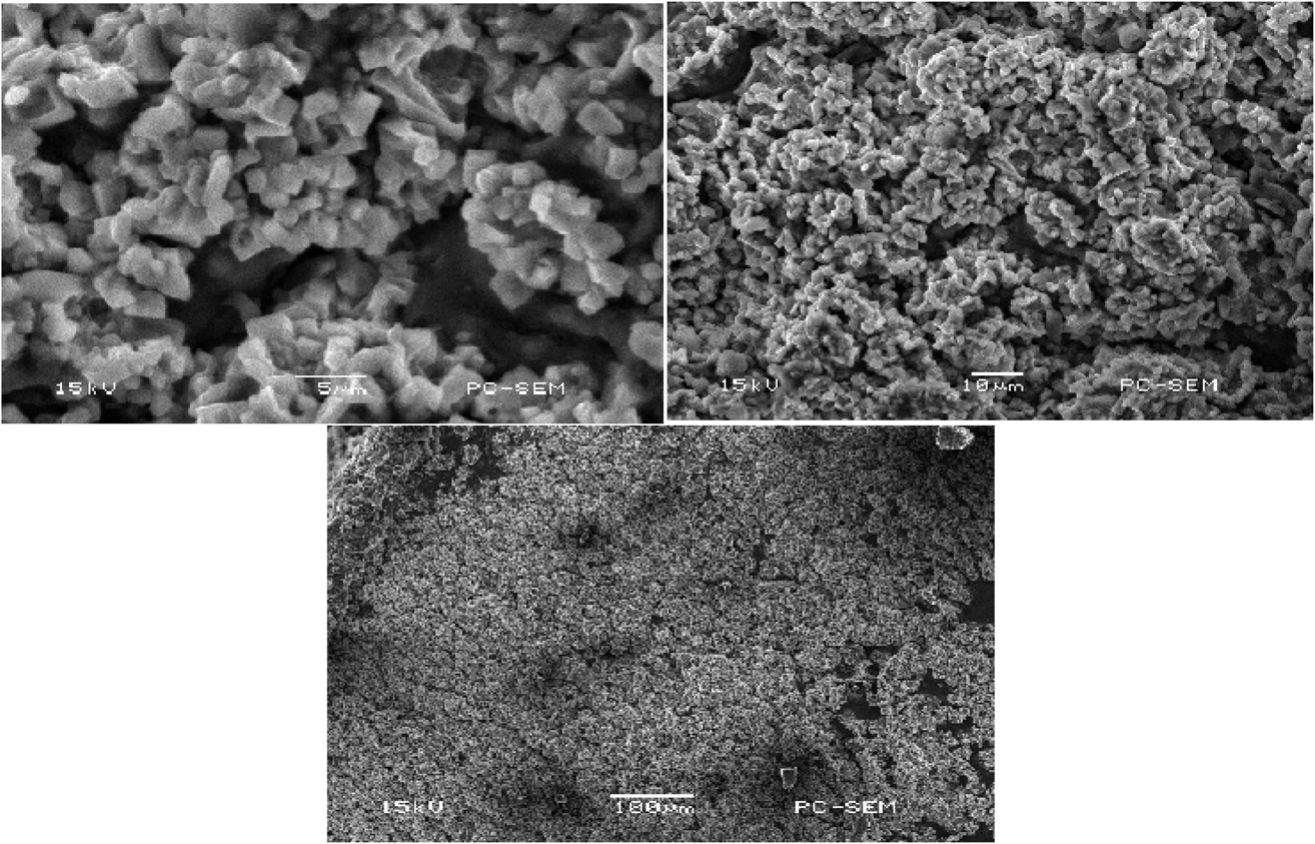
Surface morphology of drug-loaded hydrogel discs captured through SEM at varying magnifications (5 µm, 10 µm, and 100 µm), illustrating the structural architecture and porosity across different scales.

The micrographs presented in Fig. 5 reveal the compact structure and porous nature of the hydrogel formulation, indicating significant interactions between hyaluronic acid (HA) and acrylic acid (AAc). These observations suggest that successful grafting and cross-linking copolymerization occurred. The results offer valuable insights into the slower disintegration of hyaluronic acid-acrylic acid hydrogels, emphasizing the robust physical interactions between the polymer and monomer. Furthermore, the textured surface of the mesalamine-loaded discs indicates a uniform distribution of the drug within the polymeric matrix. The observed surface folds may function as sites where water can infiltrate and engage with the hydrophilic groups present within the three-dimensional polymeric structure.^25^

### 4.4. Powder X-ray diffraction (PXRD) analysis

**Fig. 6:**
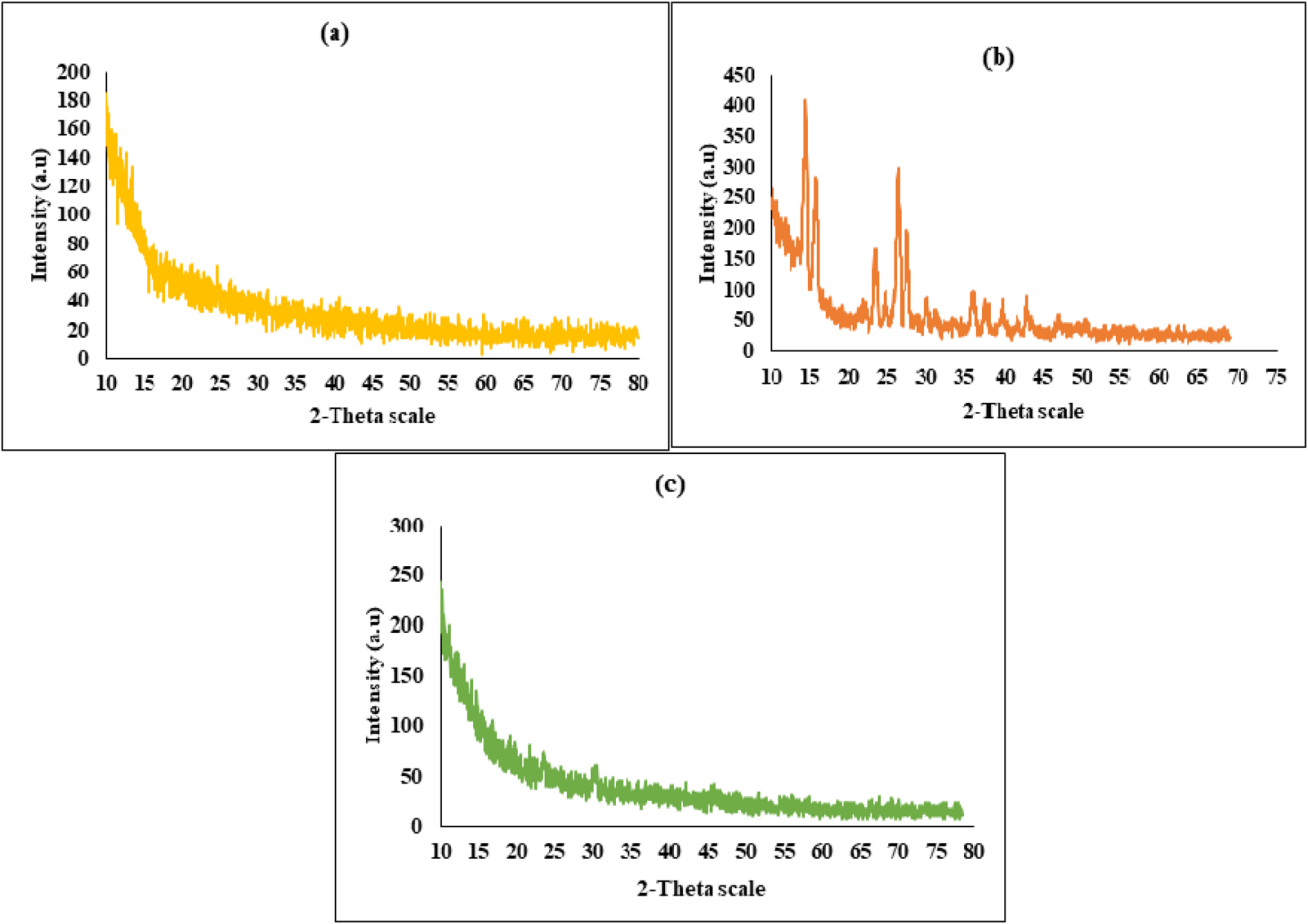
PXRD pattern of the polymer (a), drug (b), and drug-loaded hydrogels (c).

Powder X-ray diffraction (PXRD) was employed to assess the crystalline or amorphous characteristics of the drug, polymer, and polymeric network. Amorphous materials typically display increased solubility, a property that is especially beneficial for drug delivery systems designed for BCS class IV drugs, which necessitate an amorphous state to improve water solubility.^33^ The crystalline structure of mesalamine is depicted as prominent and strong peaks being detected by PXRD analysis. The drug's high crystallinity is indicated by peaks at 2*θ* = 14.52°, 15.96°, 26.52°, and 27.68°. Conversely, the PXRD diffractogram of hyaluronic acid (HA) exhibited well-defined crystalline peaks at 2*θ* values of 10.08°, 13.24°, 18.32°, 24.76°, 31.12°, and 32.40°, indicating its semi-crystalline nature. However, in the case of drug-loaded hydrogel formulations, the characteristic diffraction peaks corresponding to individual reactants and the incorporated drug were no longer observable, implying successful encapsulation and a transition toward an amorphous or molecularly dispersed state within the hydrogel matrix. This disappearance suggests that the amorphous nature of the polymeric network effectively suppressed the prominent peaks of the encapsulated drug. The observed reduction in peak intensity further confirms the successful loading of the drug into the hydrogels. Amorphous systems, with their higher molecular mobility and increased intermolecular energy compared to crystalline forms, provide a more favorable environment for enhancing various properties, such as solubility and dissolution rate.^27^ Although the incorporated drug molecules maintained their chemical identity within the hydrogel matrix, a notable reduction in their crystalline characteristics was observed. This decline in crystallinity can be attributed to the spatial confinement of drug moieties within the polymeric network, which likely disrupted regular lattice arrangements. The entrapment of drug molecules within the hydrogel pores resulted in diminished electrostatic interactions and attenuated intermolecular bonding forces, thereby promoting a transition toward an amorphous dispersion (Fig. 6).^34^

### 4.5. Sol–gel fraction

In polymeric hydrogel systems, not all components undergo complete crosslinking during the polymerization process. The uncrosslinked, soluble portion that remains in the matrix is referred to as the ‘sol fraction,’ whereas the portion that forms a stable, three-dimensional network through effective crosslinking is known as the ‘gel fraction.’ This distinction is essential for evaluating the efficiency of the crosslinking reaction and the overall structural stability of the hydrogel. The sol fraction represents the relatively minor portion of the hydrogel matrix that remains uncrosslinked, typically resulting from the use of excess polymeric or monomeric components during synthesis. This occurs due to the limited availability of reactive functional groups or steric hindrance, which prevents complete polymer network formation during the crosslinking process.^35^ To assess the impact of hyaluronic acid (HA), acrylic acid (AAc), and methylene bisacrylamide (MBA) on the sol and gel fractions of the hydrogel, discs from each formulation were weighed and subjected to Soxhlet extraction. As outlined in Table 2, a progressive increase in gel fraction was observed across formulations HA-1 to HA-3, rising from 74% to 78% in response to the elevated concentration of hyaluronic acid (HA). This enhancement is primarily attributed to the greater availability of functional moieties—specifically, carboxyl and hydroxyl groups—on the polymer backbone, which results in an increased number of potential reactive sites during crosslinking. Similarly, in formulations HA-4 to HA-6, the gel fraction markedly increased from 74% to 90% with the rising levels of acrylic acid (AAc). This trend is indicative of enhanced reactivity due to the abundance of carboxylic groups in AAc, which promotes the formation of a more robust and interconnected polymeric network. Furthermore, the formulations HA-7 to HA-9, which incorporated increasing concentrations of methylene bisacrylamide (MBA), demonstrated a modest yet consistent elevation in gel content from 74% to 77%. This increment is likely the result of an augmented crosslinking density facilitated by the additional bifunctional MBA molecules, which improve network stability and structural integrity^36^ Noreen. S. and colleagues developed pH-sensitive chemically crosslinked block copolymers and ended up coming the same insights we likewise reached.^31^

**Table 2:** Sol fraction and gel fraction of hydrogels

Formulation code	Weight before extraction (g)	Weight after extraction (g)	Sol fraction	Gel fraction
HA-1	0.69	0.55	25.45	74.54
HA-2	0.53	0.43	23.25	76.74
HA-3	0.55	0.45	22.22	77.78
HA-4	0.53	0.43	23.25	76.74
HA-5	0.67	0.57	17.54	82.46
HA-6	0.85	0.77	10.39	89.61
HA-7	0.53	0.43	23.25	76.74
HA-8	0.71	0.58	22.41	77.58
HA-9	0.62	0.51	21.57	78.43

### 4.6. Swelling studies

Substantial swelling plays a crucial role in the release of drugs from hydrogels, underscoring its significance in drug distribution within polymeric networks. Swelling behavior was examined in simulated gastric fluid (SGF) at pH 1.2 and simulated intestinal fluid (SIF) at pH 7.4. The study assessed the impact of pH as well as varying concentrations of polymer, monomer, and cross-linker on swelling characteristics. The hydrogels demonstrated markedly increased swelling at pH 7.4 compared to pH 1.2. This enhanced swelling is attributed to the deprotonation of carboxyl groups, which form carboxylate anions (COO−), leading to increased electrostatic repulsion among the anions and subsequent network expansion. Additionally, osmotic pressure further contributes to the swelling observed in SIF by intensifying the repulsion between deprotonated carboxyl groups. The observed enhancement in hydrogel swelling at pH 7.4 (simulated intestinal fluid, SIF) in comparison to pH 1.2 (simulated gastric fluid, SGF) can be attributed to the elevated solvation potential of ionised carboxylate groups in alkaline or neutral aqueous environments. These anionic moieties undergo extensive electrostatic repulsion and osmotic swelling due to their higher degree of ionisation under such conditions, unlike non-ionic or protonated counterparts which remain in a collapsed state under acidic pH, thereby significantly limiting the extent of water uptake and network expansion.^37^

Hydrogels were synthesised using systematically varied concentrations of hyaluronic acid (HA) as the polymer and acrylic acid (AAc) as the monomer to critically evaluate the influence of polymeric and monomeric content on the resultant swelling dynamics. An upward trend in swelling capacity was observed across formulations HA-1 to HA-3, corresponding with incremental increases in HA concentration. This enhancement in swelling behaviour can be attributed to the greater availability of hydrophilic functional groups—namely hydroxyl and carboxyl moieties—within the polymer matrix, which facilitates increased water uptake and network expansion through hydrogen bonding and osmotic effects. This increase in swelling can be attributed to the higher number of hydrophilic groups in the cross-linked network. As the number of these hydrophilic groups increased, the swelling media diffused more effectively into the polymeric network, resulting in enhanced swelling. Additionally, higher polymer concentrations provided more functional units for acrylic acid grafting, further contributing to the increased swelling tendency. Comparable results have been observed in earlier research, where swelling also exhibited an increase with higher concentrations of β-cyclodextrin ^27^ Dynamic swelling levels for any particular AAc concentration rise with pH. This response was more pronounced at higher pH values, as expected, due to the availability of more ionizable functional groups (carboxylic groups) in AAc. When the pH of the gel exceeds its p*K*_a_, ionization of the AAc functional groups occurs, resulting in increased hydrophilicity. If the concentration of acrylic acid was higher, the ionic interaction between acrylic acid and the polymer network was stronger. At lower pH, the linked carboxylic groups generate inter- and intramolecular H-bonding, and the hydrogel network creates fewer empty spaces for water molecules, making swelling less noticeable. At higher pH, however, the dissociation of the carboxylic groups along the network chains increases the electrostatic repulsion between the dissociated carboxylate ions (–COO^−^), which was the fundamental driving factor for network expansion. The network's expansion maximizes the free space between network chains available for water molecules, resulting in a rise in the degree of swelling.^38^

**Fig. 7:**
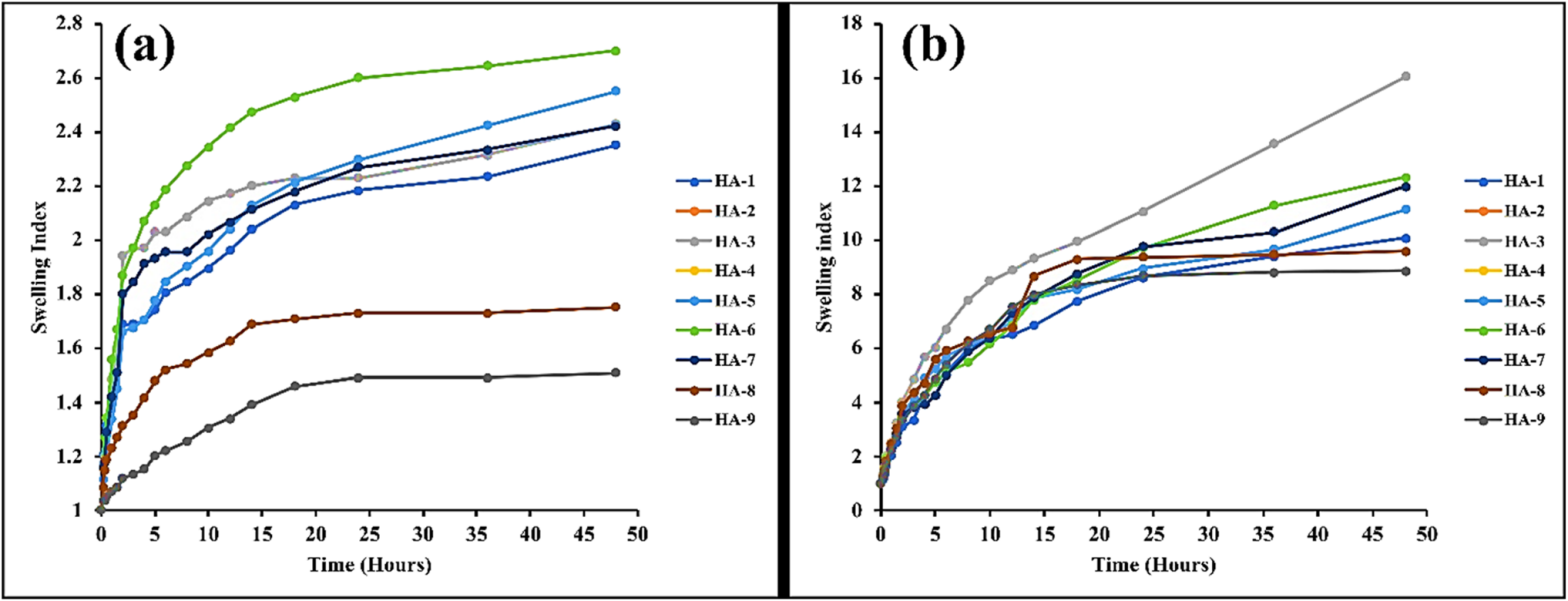
Swelling characteristics of formulated hydrogels in pH 1.2 (a) and 7.4 (b).

As the concentration of methylene bisacrylamide (MBA) increased, the swelling of the hydrogels decreased. This is because MBA creates a more densely packed polymeric network with smaller pores, reducing the porosity and consequently the swelling capacity of the hydrogels.^39^ A reduction in the pore size within the hydrogel network was observed as the cross-linker concentration increased, which consequently impeded the penetration and diffusion of the swelling medium into the polymer matrix. This restricted diffusion limited the mobility and relaxation of the polymeric chains, resulting in a diminished swelling capacity. Consequently, formulations with higher methylene bisacrylamide (MBA) content exhibited a marked decrease in swelling behavior. These observations align well with previously reported studies, which similarly documented an inverse correlation between cross-linker concentration and hydrogel swelling, attributing this effect to the increased network density and restricted polymer chain flexibility (Fig. 7).^40^

### 4.7. Polymer volume fraction

The results revealed that the polymer volume fraction increased with increasing MBA concentration while being reduced with rising hyaluronic acid and acrylic acid proportions. The related Table 3 displays the average values of the three consecutive study runs. The polymer volume fraction was greater at pH 1.2 compared to pH 7.4, a difference attributed to the swelling behavior. Hydrogels demonstrated reduced swelling at the lower pH, resulting in a higher polymer volume fraction at pH 1.2. In contrast, the lower polymer volume fraction at pH 7.4 suggests that the hydrogels are capable of significant swelling and expansion at this pH.^41^ Badshah S.F. *et al.* (2022) developed porous and highly responsive cross-linked β-cyclodextrin-based nano matrices for improvement in drug dissolution and absorption and reported outcomes that were substantially similar to ours.^27^

**Table 3:** Percent water content, percent drug loaded content, and polymer volume fraction

Formulation code	Percent water content	Percent drug-loaded content	Polymer volume fraction (pH 1.2)	Polymer volume fraction (pH 7.4)
HA-1	12.3125	72.7975	0.552	0.126
HA-2	11.32 758 621	74.62 241 379	0.498	0.118
HA-3	9.3	77.08	0.426	0.108
HA-4	11.32 758 621	74.62 241 379	0.498	0.118
HA-5	11.859 375	78.140 625	0.448	0.098
HA-6	14.54 347 826	81.36 652 174	0.389	0.081
HA-7	11.32 758 621	74.62 241 379	0.498	0.118
HA-8	14.17 391 304	69.01 608 696	0.926	0.137
HA-9	13.85 106 383	64.04 893 617	0.952	0.147

### 4.8. Percent water content

Hyaluronic acid, with its inherent 5–15% water content, contributes to residual moisture in hydrogels even after complete drying. It was crucial to assess the water content of the cross-linked hydrogels to ensure precise experimental ratios. Table 3 indicates that the water content increased with higher polymer concentrations, reflecting HA's hydrophilic nature, which enhances the hydrophilicity of the polymer network and subsequently raises the water content of the hydrogels. The total water content also increased with higher monomer concentrations, while maintaining a constant polymer/cross-linker ratio did not significantly alter the water content. Conversely, higher cross-linker concentrations resulted in more rigid, cross-linked hydrogels with reduced water content compared to those with lower cross-linker ratios. The water content measurements were critical for determining the drug loading capacity, as it directly influences this parameter. Similar findings were reported by Khaliq *et al.*, who developed biomimetic hydrogel membranes using hyaluronic acid and alginate for enhanced diabetic wound healing, yielding results comparable to those observed in this study.^42^

### 4.9. Drug loading and percent drug loaded content

The post-loading method was employed for drug incorporation into hydrogels. As detailed in Table 3, drug loading was generally effective across all formulations, with the exception of HA-8 and HA-9. All hydrogels achieved drug loading rates exceeding 65%, with formulation HA-6 demonstrating the highest drug loading at approximately 80%. The reduced drug loading observed in HA-8 and HA-9 is likely attributed to the increased cross-linker concentration, which led to a more densely cross-linked network. This dense network impedes solvent diffusion and restricts swelling—both crucial factors for effective drug loading and release. Consequently, the highly cross-linked nature of HA-8 and HA-9 resulted in lower drug loading percentages.

### 4.10. *In vitro* release

Drug release studies were conducted in buffer media with pH values of 1.2 and 7.4. The results, as shown in Fig. 6, indicated that drug release was more pronounced at pH 7.4 than at pH 1.2. This difference can be attributed to the swelling behavior of the polymeric network. At acidic pH, the network did not exhibit significant swelling, which hindered efficient drug diffusion due to the compact structure. Upon transitioning the environmental pH from acidic to alkaline conditions, the polymeric hydrogel network underwent significant swelling due to ionization of functional groups, which enhanced the ingress of water molecules into the cross-linked matrix. This swelling facilitated the diffusion and subsequent release of the encapsulated drug molecules. Notably, at pH 7.4, over 50% of mesalamine was released from hydrogel formulations HA-1 through HA-7 within the initial 20-minute period, indicating a favorable dissolution profile consistent with targeted drug delivery requirements. In contrast, only 2–10% of the drug was released within 15 minutes in the acidic medium. The effective dissolution of the drug is likely related to the hydrophilic nature and swelling ability of the polymer, which allowed the hydrogel to absorb a significant amount of solvent. This, in turn, accelerated the dissolution of the polymer and lowered the interfacial tension between the drug and water, facilitating faster drug release. Additionally, this initial swelling process may have enhanced mesalamine dissolution by increasing the surface area between the drug and hyaluronic acid or by reducing mesalamine crystallinity.

The dissolution studies demonstrated that HA was effective in improving mesalamine's solubility. All formulations from HA-1 to HA-7 showed satisfactory drug release profiles. Conversely, formulations HA-8 and HA-9 demonstrated significantly limited drug release profiles, primarily attributable to the elevated concentration of cross-linking agent. The increased cross-linker content resulted in the formation of a denser and more tightly interconnected polymeric network, which consequently diminished the hydrogel's swelling capacity. This restricted swelling impeded the penetration of the dissolution medium into the hydrogel matrix, thereby obstructing the diffusion pathways for mesalamine and culminating in a markedly reduced drug release rate.

**Fig. 8:**
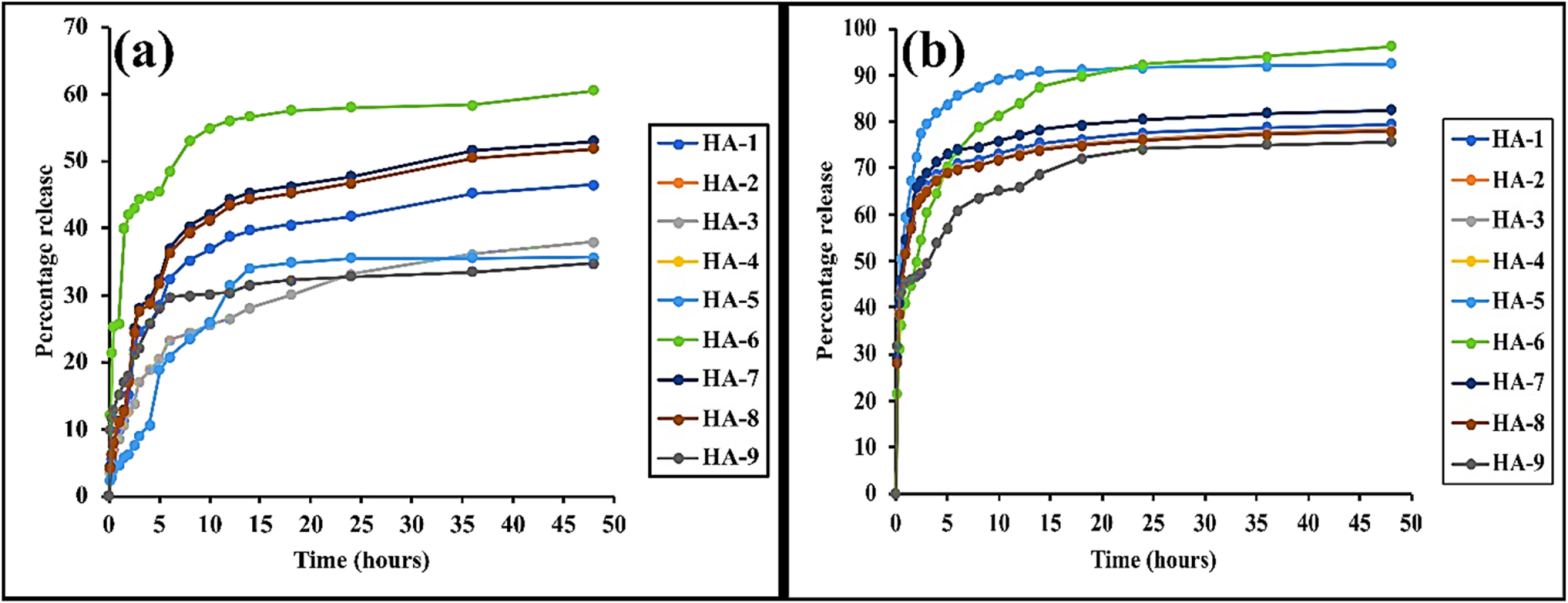
*In vitro* drug release at pH 1.2 (a) and pH 7.4 (b).

The *in vitro* drug release profiles of the synthesized hydrogels were compared to those of Asacol® tablets, which are coated with Eudragit S and release mesalamine in the terminal ileum and colon at a pH of 7 or higher. The standard adult dosage of Asacol® involves taking 2.4 grams daily for six weeks. The comparison aimed to evaluate the release behavior and effectiveness of the hydrogels in comparison to the commercially available treatment. In comparison, the drug release from the hydrogel formulations demonstrated a more sustained release profile, particularly in alkaline dissolution media, suggesting prolonged drug delivery. In contrast, the Asacol® tablet exhibited rapid release, with the majority of the drug being released within 20 minutes in a dissolution medium at pH 7.4. This rapid release resulted in a more immediate elimination of the drug from the targeted site, unlike the gradual release observed from the synthesized polymeric hydrogels (Fig. 8).

### 4.11. Biocompatibility evaluation

To assess the biocompatibility and safety profile of the newly developed hydrogel carrier system, a comprehensive toxicity evaluation was conducted using healthy rabbits. Throughout the experimental period, key physiological and behavioral parameters including body weight, food and water intake, and potential clinical signs such as fever, diarrhea, and any ocular or dermal toxicity were meticulously monitored. The results, summarized in Table 4, revealed no statistically significant differences between the control and treated groups in terms of body weight fluctuations, water consumption, or food intake, thereby indicating a favorable safety profile of the hydrogel formulation under the tested conditions. Moreover, the results of hematological analysis of rabbits' blood and biochemical analysis of kidney, liver, and lipid profiles have been depicted in Table 5 and 6. No significant changes were observed between the results of the control group and treated group.

**Table 4:** Clinical findings^a^

Study parameters	Group A (control)	Group B (treated)
Clinical symptoms	Not observed	Not observed
**Body Mass (kilograms)**		
Baseline measurement	1.88 ± 0.11	1.94 ± 0.32
Day 1 post-treatment	1.88 ± 0.02	1.94 ± 0.10
Day 7 post-treatment	1.91 ± 0.06	1.98 ± 0.04
Day 14 post-treatment	1.92 ± 0.09	2.02 ± 0.02

**Fluid consumption (milliliters)**		
Pretreatment	164.28 ± 1.18	177.16 ± 0.21
Day 1 post-treatment	175.75 ± 1.13	188.27 ± 1.34
Day 7 post-treatment	195.18 ± 3.21	203.42 ± 1.07
Day 14 post-treatment	204.43 ± 3.93	208.21 ± 3.54

**Nutritional intake (grams)**		
Pretreatment	74.15 ± 1.31	72.37 ± 1.48
Day 1 post-treatment	75.65 ± 1.10	73.98 ± 1.01
Day 7 post-treatment	77.87 ± 1.52	74.76 ± 1.23
Day 14 post-treatment	79.76 ± 1.29	76.21 ± 1.11
Skin irritation assessment	Not seen	Not seen
Ocular toxicity evaluation	Absent	Absent
Mortality rate	Nil	Nil

a Results represent the mean ± S.D.

**Table 5:** Results of hematological analysis of rabbits' blood^a^

Parameters	Group A (control)	Group B (treated)
Hemoglobin (g dL^−1^)	11.85	12.12
pH	7.15 ± 0.18	7.18 ± 0.12
White blood cells (×10^9^ L^−1^)	5.5 ± 0.22	6.8 ± 0.28
Red blood cells (×10^6^ per µL)	4.91 ± 1.06	4.99 ± 1.15
Platelets (×10^9^ L^−1^)	4.71 ± 0.12	4.66 ± 0.32
Monocytes (%)	3.68 ± 0.21	3.69 ± 0.49
Neutrophils (%)	51.39 ± 2.32	51.98 ± 2.11
Lymphocytes (%)	43.97 ± 3.15	44.21 ± 3.19
Mean corpuscular volume (%)	63.71 ± 2.18	66.19 ± 1.97
Mean corpuscular hemoglobin (pg per cell)	23.61 ± 0.64	25.02 ± 0.59
Mean corpuscular hemoglobin conc. (%)	31.41 ± 1.23	33.98 ± 1.19

a Results represent the mean ± S.D.

**Table 6:** Biochemical analysis of kidney, liver, and lipid profiles

Biochemical analysis	Group A (control)	Group B (treated)
ALT (IU/L)	136.16 ± 1.42	146.15 ± 1.26
AST (IU/L)	37.72 ± 3.11	39.94 ± 2.63
Urea (mmol L^−1^)	15.98 ± 0.41	17.07 ± 1.18
Creatinine (mg dL^−1^)	1.19 ± 0.11	1.21 ± 0.32
Uric acid (mg dL^−1^)	4.21 ± 1.56	3.81 ± 1.62
Cholesterol (mg dL^−1^)	64.51 ± 1.98	62.11 ± 2.21
Triglycerides (mg dL^−1^)	62.56 ± 2.61	61.09 ± 3.61

Hematological evaluations of blood samples indicated normal results, with a slight elevation in white blood cell count observed in the group treated with the hydrogel. Although this increase fell within standard limits, it may indicate a mild immune reaction to the hydrogel.

Histopathological examination of the major organs was performed to evaluate the potential toxicity of the hyaluronic acid-acrylic acid gels. On day 14 of the experiment, the animals were humanely euthanized, and their essential organs were carefully excised and fixed in a 10% formalin solution. The collected tissue specimens were then processed and stained using hematoxylin and eosin to facilitate microscopic examination (Table 7). The histological analysis demonstrated the absence of any signs of tissue degeneration, lesions, or pathological abnormalities in organs from both the control and treated groups.

**Table 7:** Histological studies of control and treated* group

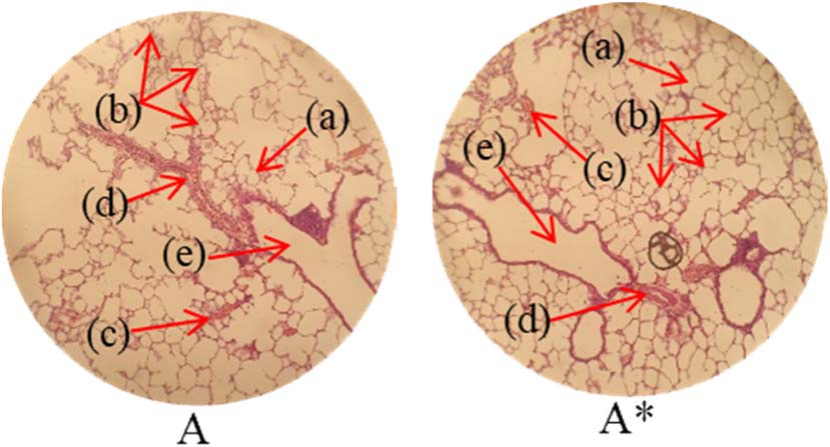	Histopathology of lungs (A, A*): Alveolus (a), alveoli (b), blood vessel (c), pulmonary vessel (d), and bronchiole (e). Heart
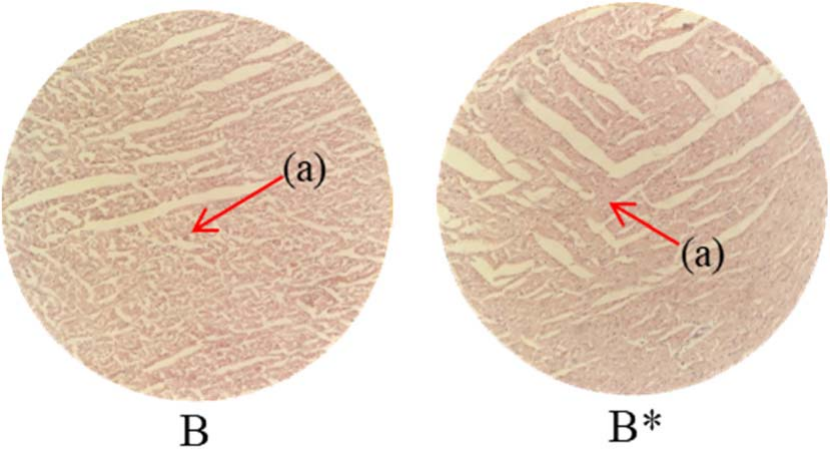	(B, B*): Cardiac muscle fibers (a). Colon
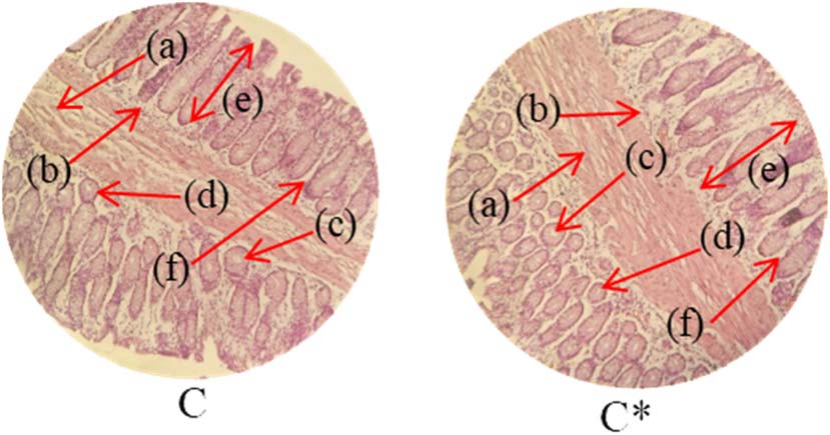	. (C, C*): Sub mucosa (a), muscularis mucosa (b), lumen of crypt (c), colonic crypt (d), mucosa (e), and lamina propria (f). Kidney
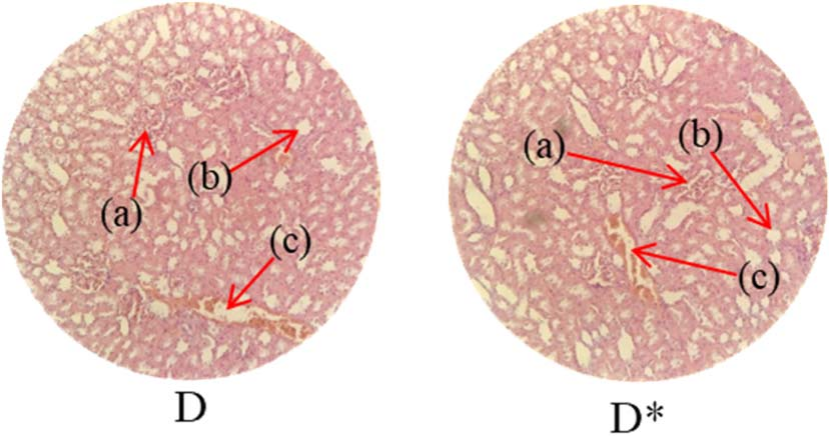	(D, D*): Glomerulus (a), renal tubules (b), and blood vessels (c). Small intestine
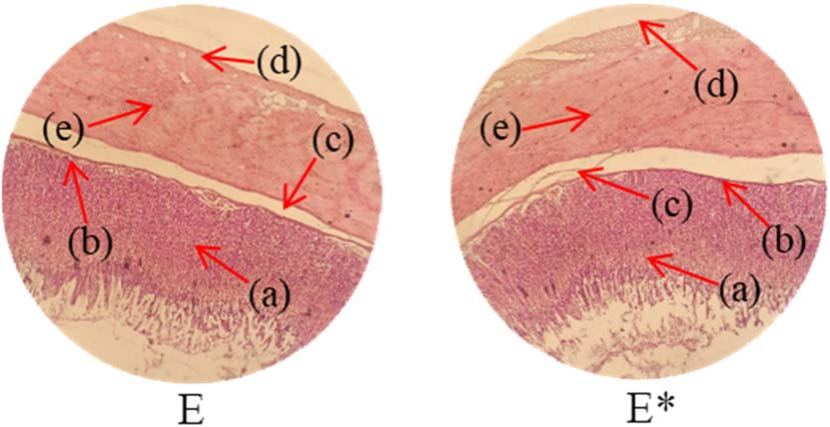	(E, E*): Small intestinal villi (a), intestinal gland (b), muscularis mucosa (c), serosa (d), and muscularis externa (e). Spleen
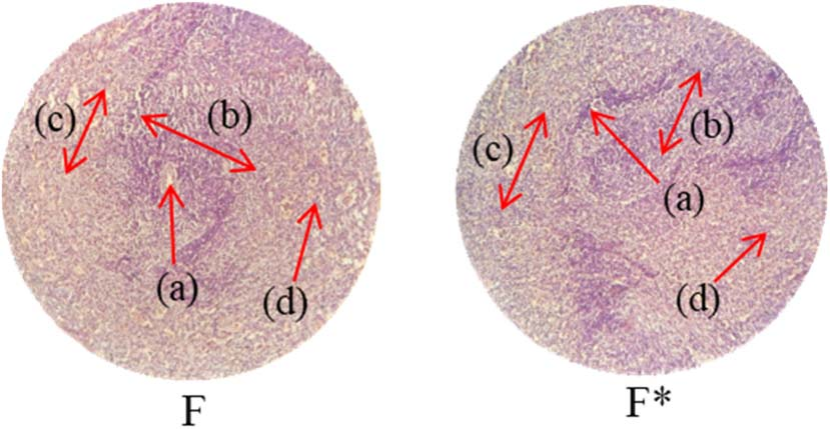	(F, F*): Central arteriole (a), white pulp (b), red pulp (c), and trabecular (d). Liver
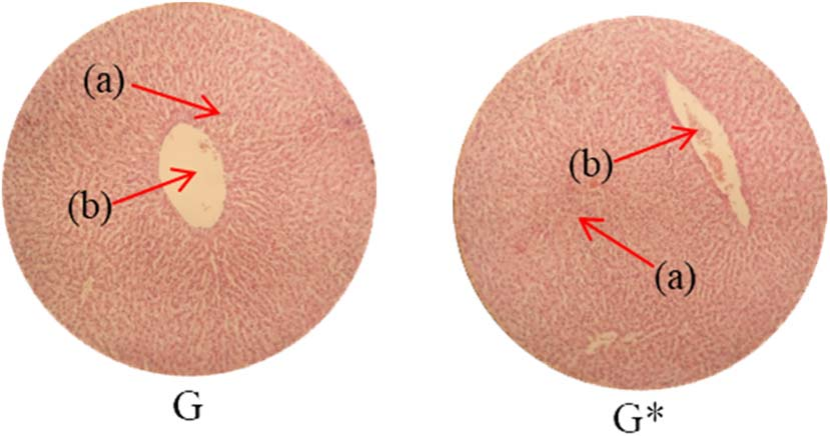	(G, G*): Plates of hepatocytes (a), and blood vessels (b)

Liver sections from both groups exhibited mild hyperplasia and localized inflammatory cell infiltration. Cardiac tissue exhibited a typical organization of cardiomyocytes, with no evidence of hypertrophy or myocardial infarction. Histopathological examination of lung tissue revealed slight edema and alveolar fluid accumulation, however, no indications of fibrosis were observed. Intestinal and colonic tissues appeared structurally intact, with preserved muscular layers and columnar epithelium, and no signs of inflammation. Kidney tissue demonstrated healthy glomeruli, Bowman's capsules, and tubules. Spleen tissue demonstrated a normal morphology, characterized by a uniform distribution of lymphocytes within the white pulp.

No notable differences were observed between the control group and the cohort treated with HA-AAc hydrogels. These findings are consistent with those reported by U. Rehman *et al.* (2022), who also developed pH-responsive hydrogels for drug delivery and observed similar results in toxicity studies conducted on rabbits.^43^

### 4.12. Stability studies

The stability studies demonstrated that the hydrogels maintained their physical integrity over 6-months, with no visible signs of deformation, discoloration, or surface cracking. FTIR spectra collected at 3 and 6 months showed no significant changes in characteristic peaks, indicating the chemical stability of the polymer network. Furthermore, the drug release profiles of 5-ASA remained consistent at each time point, suggesting that storage under accelerated conditions did not adversely affect the hydrogel's release behavior. Overall, these findings confirm that the formulated hydrogels exhibit good stability and sustained performance over the tested period.

## 5. Conclusion

The free radical polymerization technique has been successfully utilized to develop a well-organized, thermally stable hydrogel system. The effective grafting of acrylic acid onto the hyaluronic acid backbone was confirmed by Fourier-transform infrared spectroscopy, which indicated successful crosslinking during the polymerization phase. Thermogravimetric analysis data revealed that the synthesized hydrogel exhibits enhanced thermal stability compared to the unreacted hyaluronic acid and mesalamine. Scanning electron microscopy images at various magnifications revealed an irregular and porous surface morphology of the hydrogel. Swelling and *in vitro* drug release studies demonstrated that these properties were significantly higher in basic media compared to acidic conditions. Additionally, increased concentrations of hyaluronic acid and acrylic acid correlated with enhanced swelling and drug release, whereas elevated concentrations of cross-linker resulted in reduced swelling and drug release. In summary, hyaluronic acid-based hydrogels show promising potential as an alternative to traditional dosage forms for the controlled and prolonged release of mesalamine, leveraging hyaluronic acid's inherent wound healing properties. However, a key research gap remains in translating these formulations from experimental to clinical settings. Specifically, further investigation is needed to assess the biocompatibility, long-term stability, and clinical efficacy of these hydrogels in real-world applications, as well as to optimize their formulation for large-scale manufacturing and regulatory approval. Additionally, rigorous evaluation in preclinical models is necessary to confirm the efficacy and safety of the hydrogel in therapeutic settings.

## Ethical statement

The animal study involving rabbits was duly approved by the Institutional Research Ethics Committee (IREC) of the Faculty of Pharmacy, The University of Lahore, Pakistan under the reference number IREC-2023-60. All established ethical standards for the care and use of laboratory animals were strictly followed.

## Author contributions

H. L : conceptualization, investigation, methodology, roles/writing – original draft; S. F. B.: conceptualization, project administration, resources, supervision; M. U. M., M. S., G. M. E.: data curation, formal analysis, resources, writing – review & editing; K. B., E. M. A.: formal analysis, investigation, validation; O. A. A. M.: formal analysis, validation, visualization; S. K: data curation, formal analysis, visualization: S. C., H. C., A. A. M.: formal analysis, validation, writing – review & editing.

## Conflicts of interest

All authors declare that they have no conflict of interest.

## Supplementary Material

RA-016-D5RA08622A-s001

## Data Availability

All data generated or analyzed during this study are included in this published article. Supplementary information (SI) is available. See DOI: https://doi.org/10.1039/d5ra08622a.
