# Supplementary material for: pH-responsive hyaluronic acid-co-poly(acrylic acid) hydrogel scaffolds for modified release of mesalamine; characterization and biosafety evaluation
Source: RSC Adv. 2026 Jan 26;16(6):5561–77. doi: 10.1039/d5ra08622a (PMC12833727; doi:10.1039/d5ra08622a)
Supplement: RA-016-D5RA08622A-s001 [file RA-016-D5RA08622A-s001.pdf]

Supplementary Information (SI) for RSC Advances.
This journal is © The Royal Society of Chemistry 2026

pH-responsive hyaluronic acid-co-poly (acrylic acid) hydrogel scaffolds for modified release of mesalamine; characterization and biosafety evaluation

Huma Liaqat[1], Muhammad Usman Minhas[2], Syed Faisal Badshah[3*], Kashif Barkat[1,4], Samiullah Khan[5], Shivani Chopra[6], Hitesh Chopra[7], Esmael M. Alyami[8], Amira Metouekel[9], Atrsaw Asrat Mengistie[10*], Gehan M. Elossaily[12,13], Mudassar Shahid[11]

[1] Faculty of Pharmacy, The University of Lahore, Lahore, Pakistan. pharmacisthuma123@gmail.com, kashif.barkat@pharm.uol.edu.pk

[2] College of Pharmacy, University of Sargodha, Sargodha, Pakistan. us.minhas@hotmail.com

[3] Faculty of Medical and Health Sciences, Department of Pharmacy, University of Poonch Rawalakot, Azad Jammu and Kashmir, Pakistan. faisal.badshah@upr.edu.pk

[4] Faculty of Health Sciences, Equator University of Science and Technology, Masaka City, Uganda.

[5] College of Pharmacy, Margalla Institute of Health Sciences, Islamabad, Pakistan. sami_pharmacist99@hotmail.com

[6] Department of Biosciences, Saveetha School of Engineering, Saveetha Institute of Medical and Technical Sciences, Chennai - 602105, Tamil Nadu, India. shanuvashisht2730@gmail.com

[7] Centre for Research Impact & Outcome, Chitkara College of Pharmacy, Chitkara University, Rajpura, 140401, Punjab, India. chopraontheride@gmail.com

[8] Department of Biology, College of Science, King Khalid University, PO Box 960, Asir, Abha, 61421, Saudi Arabia. ealshahi@kku.edu.sa

[9] University of Technology of Compiègne, EA 4297 TIMR, 60205 Compiègne Cedex, France. amira.m@bioval-oi.com

[10] Department of Biology, Bahir Dar University, P.O.Box 79, Bahir Dar, Ethiopia. Atrsawresearcher@gmail.com

[11] Department of Pharmaceutics, College of Pharmacy, King Saud University Saudi Arabia. mahmad1@ksu.edu.sa

[12] Department of Basic Medical Sciences, College of Medicine, AlMaarefa University, Diriyah, 13713, Riyadh, Saudi Arabia. jabdelmenam@um.edu.sa

[13] Research Center, Deanship of Scientific Research and Post-Graduate Studies, AlMaarefa University, Diriyah, 13713, Riyadh, Saudi Arabia.

* Correspondence: faisal.badshah@upr.edu.pk (SFB); Atrsawresearcher@gmail.com (AAM)

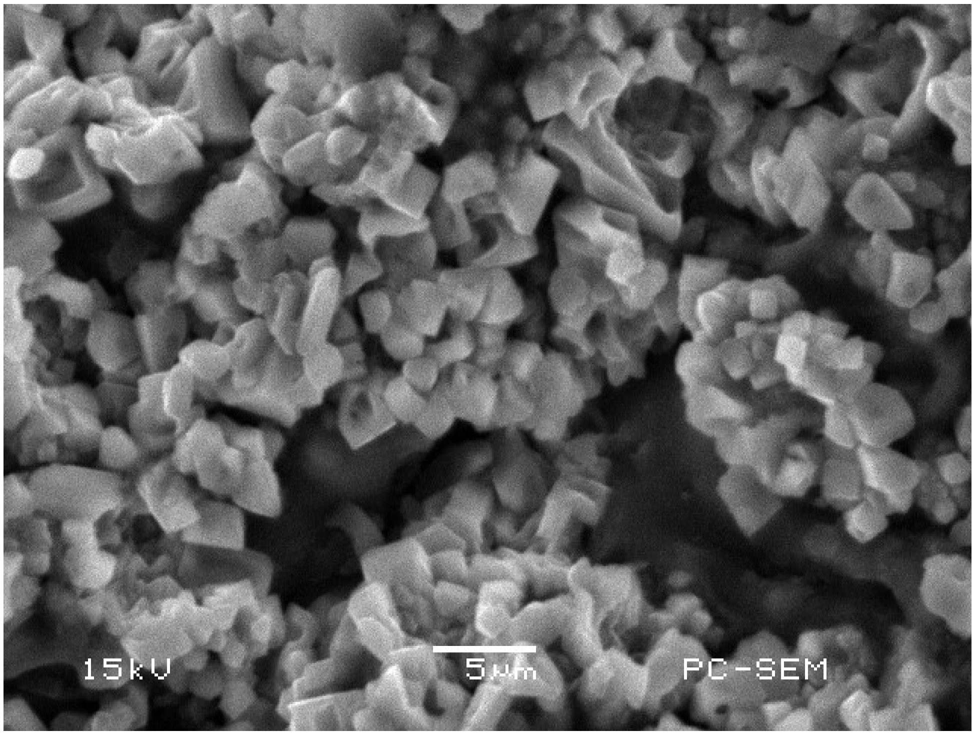

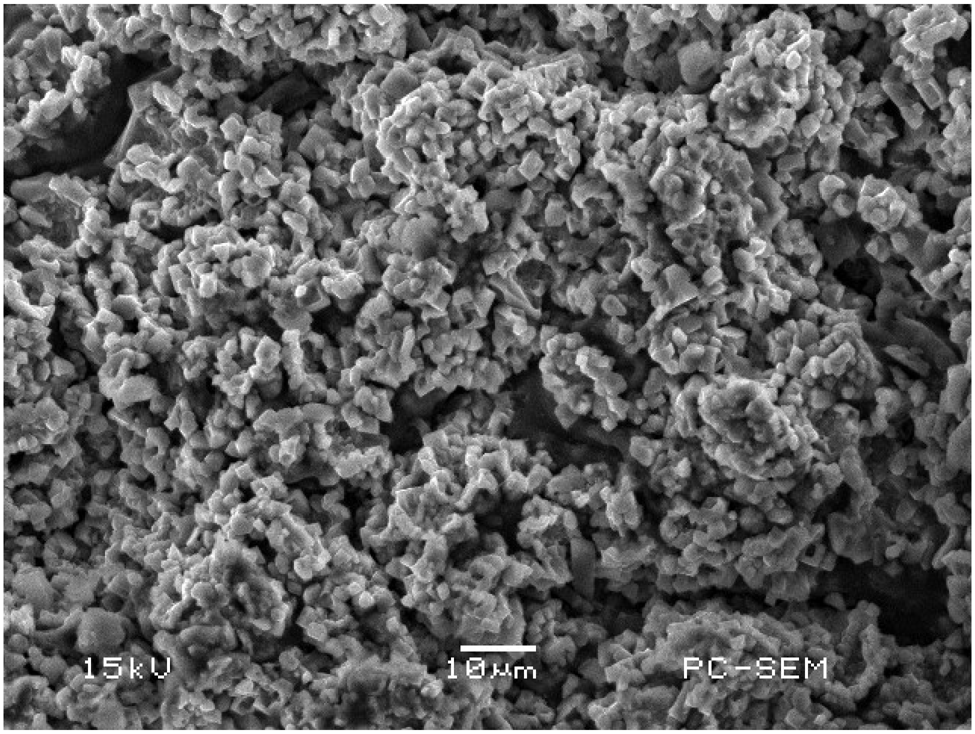

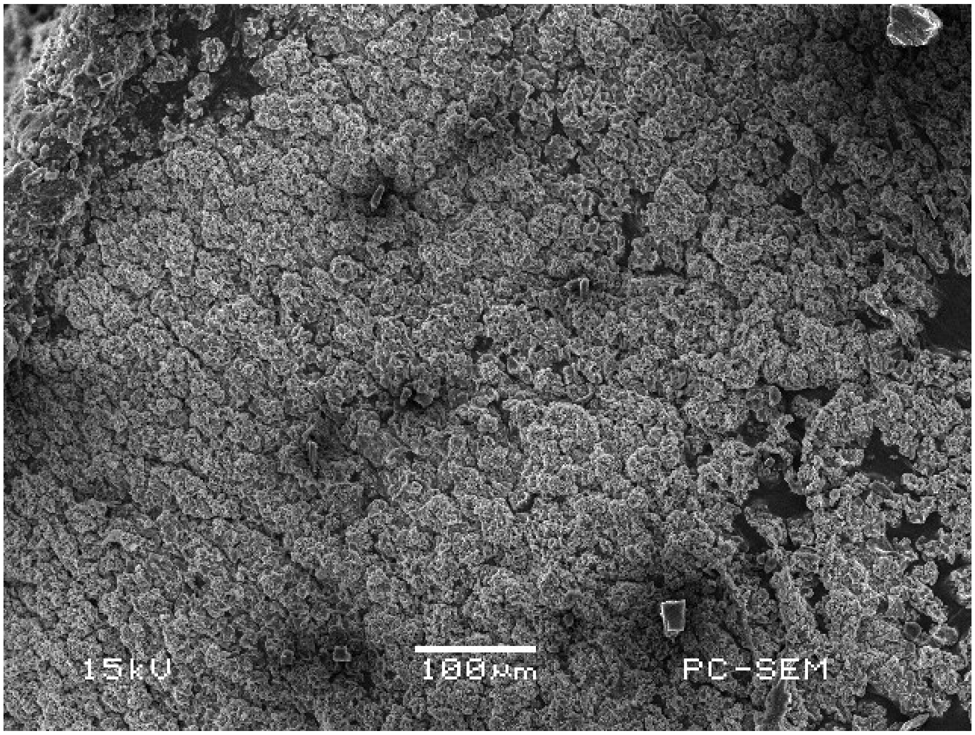

Figure S1. Surface morphology of drug-loaded hydrogel discs captured through SEM at varying magnifications (5 µm, 10 µm, and 100 µm), illustrating the structural architecture and porosity across different scales.
